# A metabarcoding approach for the feeding habits of European hake in the Adriatic Sea

**DOI:** 10.1002/ece3.4500

**Published:** 2018-10-06

**Authors:** Giulia Riccioni, Marco Stagioni, Corrado Piccinetti, Simone Libralato

**Affiliations:** ^1^ Department of Biological, Geological and Environmental Sciences University of Bologna Bologna Italy; ^2^ Chapter of Oceanography Echo Group National Institute of Oceanography and Experimental Geophysics – OGS Trieste Italy

**Keywords:** Adriatic Sea, European hake, feeding habits, *Merluccius merluccius*, metabarcoding

## Abstract

European hake (*Merluccius merluccius*) is one of the most economically important fish for the Mediterranean Sea. It is an important predator of deep upper shelf slope communities currently characterized by growth overexploitation: the understanding of hake's diet might support next generation management tools. However, all current European hake diet studies depend on the morphological identification of prey remains in stomach content, with consequent limitations. In this study, we set up a metabarcoding approach based on cytochrome oxidase I PCR amplification and Miseq Illumina paired‐end sequencing of *M. merluccius* stomach content remains and compared the results to classic morphological analyses. A total of 95 stomach contents of *M. merluccius* sampled in the North‐Central Adriatic Sea were analyzed with both the metabarcoding and morphological approaches. Metabarcoding clearly outperformed the morphological method in the taxonomic identification of prey describing more complex trophic relationships even when considering the morphological identification of 200 stomach contents. Statistical analysis of diet composition revealed a weak differentiation among the hake's size classes, confirming an opportunistic feeding behavior. All the analyses performed showed the presence of a core of shared prey among the size classes and a cloud of size‐specific prey. Our study highlights the exceptional potential of metabarcoding as an approach to provide unprecedented taxonomic resolution in the diet of *M. merluccius* and potentially of other marine predators, due to the broad‐spectrum of detection of the primers used. A thorough description of these complex trophic relationships is fundamental for the implementation of an ecosystem approach to fisheries.

## 1 INTRODUCTION

Quantitative assessment of food habits is an important aspect of fisheries management as the knowledge of both predator and prey resources can help guide management efforts aimed at increasing fish production. Accurate description of fish diet and feeding habits in aquatic environments (Chipps & Garvey, 2007) in fact provides the basis for a more comprehensive understanding of dynamics of target species by including their trophic interactions (e.g., Angelini et al., 2016; Punt, Ortiz, Aydin, Hunt, & Wiese, 2016), a basic requirement for ecosystem‐based fishery management (Möllmann et al., 2014; Pikitch et al., 2004; Zhou et al., 2010). For instance, the reconstruction of trophic links between marine fishes allows including food interactions into assessments (Punt et al., 2016) or more generally may serve as a basis for setting a balanced exploitation across trophic levels (Garcia, Rice, & Charles, 2014; Garcia et al., 2015), thereby preventing the fishing‐induced trophic level decline (Shackell, Frank, Fisher, Petrie, & Leggett, 2010). Overall, approaches based on food webs can provide a fisheries management advice based on broader and more realistic context than single species approaches (see Link, 2002; Mackinson, Deas, Beveridge, & Casey, 2009; Walters, Christensen, Martell, & Kitchell, 2005). Moreover, the study of feeding habits is necessary and useful to understand mechanisms and processes which structure and influence fish assemblages (Carlucci et al., 2018; Eriksson et al., 2011; Kotrschal & Thomson, 1986).

Nevertheless marine food webs can be extraordinarily complex with a multitude of species connected by a tangled web of predator–prey interactions. In fact omnivory is widespread in the marine environment as species have large spectrum of prey and can have large bathymetric ranges (Carpentieri, Colloca, Cardinale, Belluscio, & Ardizzone, 2005; Polunin & Pinnegar, 2002), and because typically many species undergo to important changes in feeding habits and preferences during ontogenetic growth (Belgrano, 2005). Therefore, although necessary, describing these food web structures is particularly challenging owing to data limitations. The metabarcoding approach can contribute to overcome these limitations by a better identification of trophic links.

Current studies on feeding habits (including stable isotopes), in fact, mainly relied on the morphological identification of prey remains in stomach content: this method is labor‐intensive, time expensive and depends heavily upon the skills of the taxonomist identifying semi‐digested fragments. Moreover, it precludes the identification of foods that leave no hard remains or lack diagnostic taxonomic features; thus, the contribution of some prey to the diet composition might be underestimated or neglected (Baker, Buckland, & Sheaves, 2014; Buckland, Baker, Loneragan, & Sheaves, 2017).

Conversely, recent DNA‐based approaches represent a powerful means in dietary analysis (Kress, García‐Robledo, Uriarte, & Erickson, 2015). Taxon detection from bulk samples can be achieved using PCR amplification followed by massive parallel sequencing of homologous gene regions. These short genomic DNA regions are used like unique species tag (barcodes) for specimen identification (Hebert, Cywinska, Ball, & deWaard, 2003). The obtained sequences are then compared to reference barcodes in public databases to determine similarity for taxonomic identification. This so‐called metabarcoding approach (Taberlet, Coissac, Hajibabaei, & Rieseberg, 2012) has proved to be an effective tool for characterizing the diet of predators (Deagle, Chiaradia, McInnes, & Jarman, 2010; Deagle, Kirkwood, & Jarman, 2009; Murray et al., 2011; Shehzad et al., 2012) and herbivores (Soininen et al., 2009; Valentini et al., 2009) through analysis of their feces or gut content. However, at present, metabarcoding applications in marine food webs are still limited (Albaina, Aguirre, Abad, Santos, & Estonba, 2016; Berry et al., 2015; Leray, Meyer, & Mills, 2015; Leray et al., 2013).

We tested the metabarcoding approach using an important predator species and one of the most economically important demersal fisheries resource for the Mediterranean which is the European hake (*Merluccius merluccius*, Linnaeus 1758, Figure 1). The European hake is a nektobenthic predator of communities inhabiting the Mediterranean shelf and upper slope, showing a very wide depth range (20–1,000 m) throughout the Mediterranean Sea and the Northeastern Atlantic (Carpentieri et al., 2005). *Merluccius merluccius* is a predator species of high commercial interest for the Mediterranean fisheries with 20,345 t of catch in 2014 (8,735 t by Italian fisheries, source FAO Regional capture fisheries statistics) and represents one of the main resources for Mediterranean trawl fisheries (summing up to 1.6% of total Mediterranean and Black Sea average catches in the 2000–2013 period; FAO, 2016). According to recent assessments (STECF, 2015), this species is heavily overfished in all northern Mediterranean countries. It is expected that the dynamics of this voracious predator affect other species in the ecosystem through predation control of its prey. In order to know implication of fishing management measures, therefore, it is important to have a good understanding of its food preferences.

**Figure 1:**
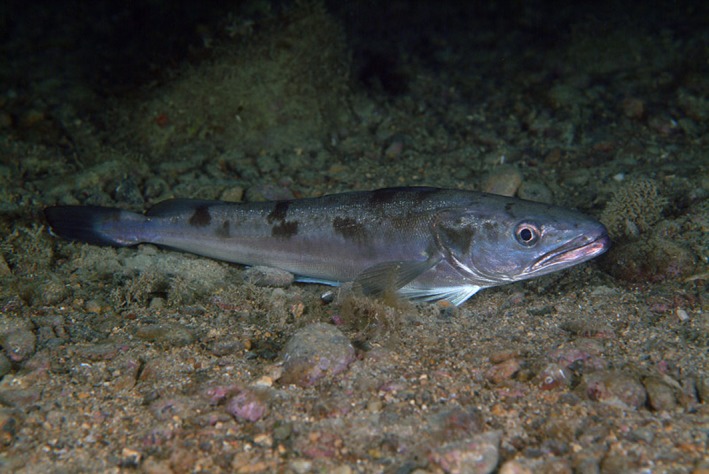
European hake picture. The European hake is a nektobenthic predator of communities inhabiting the Mediterranean shelf and upper slope (image courtesy Stefano Guerrieri).

Although the feeding habits of European hake have been described widely in the Mediterranean (Bozzano, Sardà, & Ríos, 2005; Carpentieri, Colloca, & Ardizzone, 2008; Carpentieri et al., 2005; Cartes, Rey, Lloris, & De Sola, 2004; Froglia, 1973; Papaconstantinou & Caragitsou, 1987; Sartor, Carlini, & De Ranieri, 2003; Stagioni, Montanini, & Vallisneri, 2011; Ungaro, Mannini, & Vrgoč, 2003), they show important differences justified by the opportunistic behavior of this predator. According to these works based on the morphological identification of prey remains in stomachs, adult hakes feed mainly on fish (anchovies, pilchard and gadoid species) and squids whereas the juveniles (<160 mm) feed mainly on crustaceans (preferentially euphausiids and amphipods) in the Mediterranean.

In this study, we set up a metabarcoding approach based on cytochrome oxidase I (COI) PCR amplification of stomach content remains of five size classes of *M. merluccius* of the Adriatic Sea (Mediterranean Sea). The North‐Central Adriatic Sea is the largest shelf area of the Mediterranean where maximum depth ranges between 75 and 100 m, with the exception of the Pomo/Jabuka Pit (200–260 m). Within the Mediterranean, the Adriatic basin represent an ideal study area because European hake spends its entire life cycle, including the spawning season, in the basin and in the Pomo/Jabuka Pit are located the nursery areas of this species (FAO resource, http://www.faoadriamed.org/italy/html/Species/MerlucciusMerluccius.html#C).

We compared the efficiency of this DNA‐based method to the classical morphological analysis to quantify dietary richness, diet composition, and potential overlap among the size classes. Moreover, using a mock positive control, we evaluated metabarcoding efficiency in species identification and the possible range of OTUs (Operational Taxonomic Units) number for each individual sample.

## 2 MATERIALS AND METHODS

### 2.1 Sampling strategy

**Figure 2:**
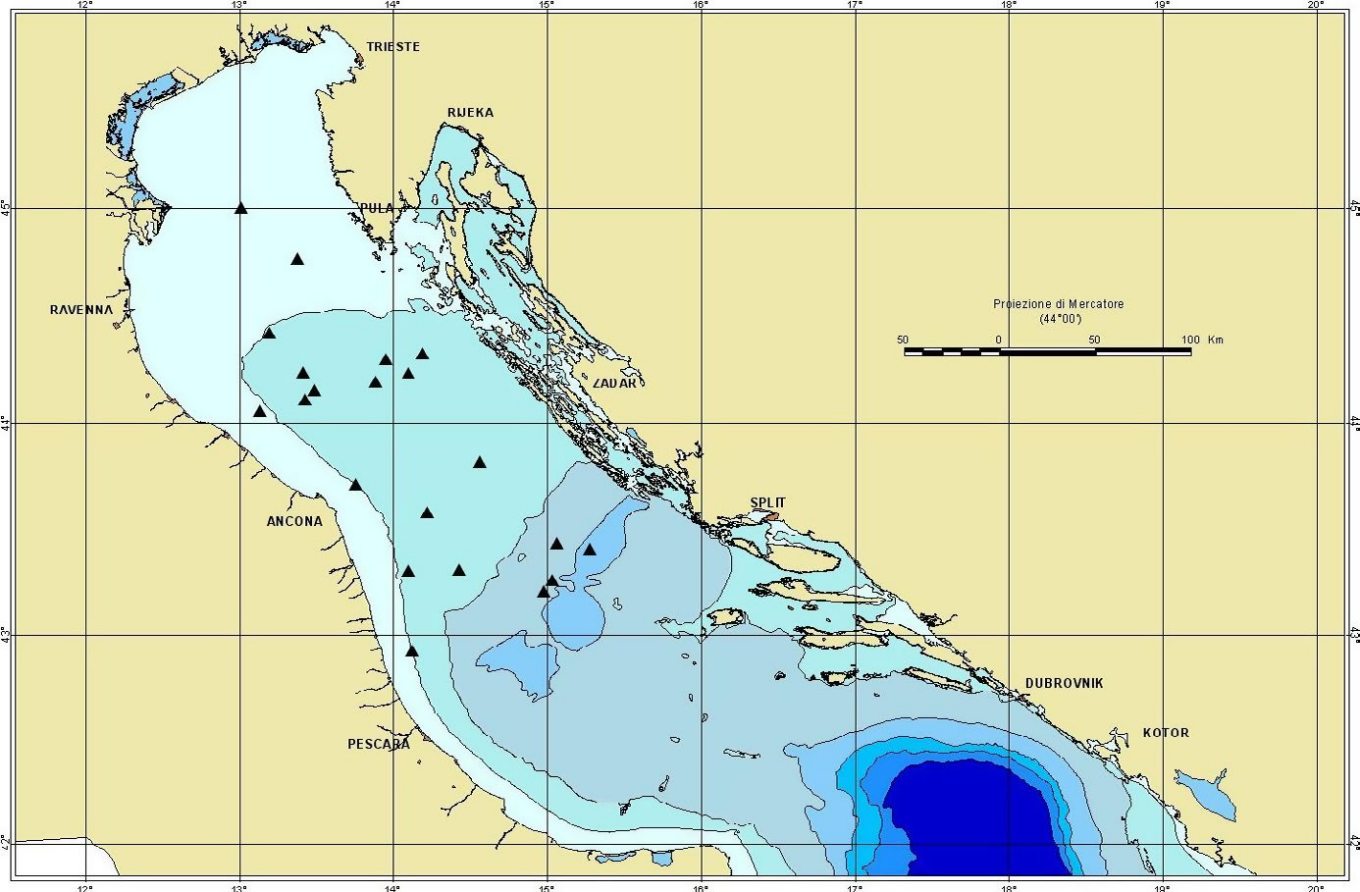
Map of the sampling hauls in the Adriatic Sea. Further details can be found in Supporting Information Table S1. This map was created using ArcViewGIS version 3.2a (https://geonet.esri.com/thread/36365). Image courtesy of Chiara Manfredi. The Adriatic cartography used is freely available at http://www.faoadriamed.org/html/adr_inf_centre.html#cart

European hake specimens were collected between 32 and 143 m depth along the coast of the Adriatic Sea (Northeast Mediterranean) from the Gulf of Trieste to Pomo/Jabuka pit (Figure 2 and Supporting Information Table S1) within the framework of International Bottom Trawl Survey in the Mediterranean (MEDITS) cruises during the campaign of the year 2014. Nineteen individuals each of the 5 size class for a total of 95 individuals were selected for the metabarcoding and morphological analyses. The five size classes (size class 1 = TL 120–149 mm, size class 2 = TL 150–199 mm, size class 3 = TL 200–249 mm, size class 4 = TL 250–299 mm, size class 5 = TL ≥300 mm) were defined on the basis of previous results, keeping in mind size distribution by bathymetric and geographical strata, abundance and feeding habits (Stagioni et al., 2011). The stomachs were dissected and preserved in 95% ethanol at −20°C.

Although the direct comparison between metabarcoding and morphological approach was carried out on the 95 stomachs, results from morphological analysis carried on additional 105 hake's stomachs collected in the same area and same campaign were also used for highlighting outperformances of the molecular approach.

### 2.2 DNA molecular analysis

*Merluccius merluccius* prey were identified to the lowest possible taxonomic level, counted, and weighed to the nearest 0.1 mg after removal of surface water by blotting paper. After morphological identification, all the content of 95 stomachs was homogenized and 100 μl of the homogenate were used for the molecular analysis. Moreover, some common prey of European hake was collected (Supporting Information Table S2) and approximately 25 mg of muscle was dissected and used for single‐species DNA extraction. These samples were used to set up PCR conditions in order to amplify at least the most common prey of *M. merluccius*. Furthermore, the obtained DNA was quantified with Qubit fluorometer (dsDNA HS Assay kit; Invitrogen, Supporting Information Table S2), diluted 1:100 and 1 μl of each was used to create a mock positive control (mixture of known species) to be amplified through PCR, sequenced in the same Illumina Miseq lane of the stomach samples and analyzed using the same approach as the stomach contents. All DNAs were extracted using a commercial kit (DNeasy Blood & Tissue Kit, QIAGEN). Samples were processed in small batches representing five size classes of *M. merluccius* (19 specimens each) with an extraction blank to monitor for potential cross‐contamination in a separated room designated to conduct molecular diet analyses. To avoid cross‐contamination, dissection tools were flame sterilized between individuals and lab surfaces were decontaminated with bleach. The primer pair selected for DNA amplification (mlCOIintF and jgHCO2198, Leray et al., 2013) was analyzed using the ecoPCR software, an in silico PCR program that allows imperfect matches between each barcode primer and its binding site to mimic in vitro PCR (Ficetola et al., 2010). The ecoPCR analysis was performed to evaluate the amplification efficiency on the two major taxonomic groups representing European hake prey (invertebrate and vertebrate subphylum, EMBL database 124 release). PCR amplification was performed in two replicates in a total volume of 25 μl with 0.75 μl of 10 μM of each forward and reverse primers, 0.2 μl of AmpliTaq Gold^®^ DNA Polymerase (ThermoFisher) 5 U/μl, 2 μl of 25 mM Mg2+, 0.5 μl of 10 mM dNTP, 1 mg/ml BSA and 3 μl of genomic DNA. We used a “touchdown” PCR profile (Leray et al., 2013 modified) to minimize the probability of nonspecific amplifications. We carried out 16 initial cycles: denaturation for 10 s at 95°C, annealing for 30 s at 62°C (−1°C per cycle) and extension for 60 s at 72°C, followed by 25 cycles at 46°C annealing temperature (−0.2°C per cycle). All PCRs included no‐template controls, and the products were checked on 1.5% agarose gels. For DNA amplification and library preparation, 20 tagged primers were used (primers Leray et al., 2013 modified). All the tagged amplicons (313‐bp plus tag) were purified with Sera‐Mag SpeedBeads (GE Healthcare Life Sciences) purification protocol (Rohland & Reich, 2012), quantified with Qubit fluorometer and pooled in equimolar concentration. Illumina MiSeq sequencing (2 × 250) was performed by Fasteris SA (Fasteris SA, 1228 Plan‐les‐Ouates, Switzerland) following Metafast protocol (PCR‐free protocol for library preparation) and approximately 17 × 10^6^ paired‐end sequences were obtained.

### 2.3 Bioinformatic and statistic methods

Sequence demultiplexing, quality control, PCR, and sequencing error filtering were performed using OBITools software (Boyer et al., 2016; http://metabarcoding.org/obitools/doc/welcome.html). The *illuminapairedend* command was used to perform a micro‐assembly of paired‐end reads. Sequences with Illumina fastq quality scores <30 across the head, tail, or total length of the sequence were discarded. We used *ngsfilter* command to assign the reads to each sample through barcode identification (14 × 10^6^ sequences). Only the sequences longer than 100 bp were retained and dereplicated using *obiuniq* command. We further filtered the sequences and those with count <10 were discarded; moreover, the *obiclean* command were used to detect the potential PCR errors selecting only sequences with the “*head”* status and abundance higher than 0.05%.

Two different approaches have been used to evaluate the *M. merluccius* diet composition from the metabarcoding data: (a) sequence occurrence (i.e., presence/absence), (b) OTUs (Operational Taxonomic Units) Relative Abundance (ORA), the proportion of unique OTUs in a sample divided by the final number of OTUs (after bioinformatic processing) in that sample. We used ORA data to evaluate if inferences based on relative abundance differed from those obtained using occurrence data and to provide a proxy of the relative amount of marine organisms in *M. merluccius* diet. Most of our inferences, however, were based on occurrence data because of the semi‐quantitative nature of metabarcoding analysis (Pompanon et al., 2012; Thomas, Jarman, Haman, Trites, & Deagle, 2014).

For taxonomic assignments, we performed BLASTn (Zhang, Schwartz, Wagner, & Miller, 2000) searches of OTU representative sequences against full GenBank database (November 2015). We used BLAST algorithm optimized for very similar sequences (megablast) on the nucleotide collection (nr/nt) that includes all GenBank + EMBL + DDBJ + PDB sequences restricting the search to sequences with >95% of similarity. Moreover, we accepted a species level match when similarity to the reference barcode was ≥97%. Sample‐based Mao Tau rarefaction curves and nonparametric species richness estimators were computed in EstimateS (Colwell, 2006). Inter‐size class variability was measured using Bray‐Curtis dissimilarities (Oksanen et al., 2016), which range from 0 (complete overlap) to 1 (complete nonoverlap), to compute pairwise community distance matrices and examine differences in beta diversity. Patterns of sample dissimilarity were visualized using PCoA. A non‐parametric analysis of similarity (R‐vegan function *anosim*; 1,000 Monte Carlo permutations) was used to test the null hypothesis of no difference in species composition among samples. Moreover, to refine this analysis, we performed a permutational (per)MANOVA test that can accommodate both categorical and continuous predictor variables (R‐vegan function *adonis*, 1,000 permutations). All these analyses were carried out using the *vegan* package (Oksanen et al., 2016) in R (R Development Core Team, 2015). To further explore relative occurrence data, we applied generalized linear models (GLM) using the *mvabund* R package. Many commonly used multivariate analyses (e.g., PERMANOVA, ANOSIM, CCA, RDA, etc.), are indeed “distance‐based analyses.” This means the first step of the analysis is to calculate a measure of similarity between each pair of samples, thus converting a multivariate dataset into a univariate one. Their statistical power is very low, except for variables with high variance. GLM do not suffer for this weakness, thus was used to the multivariate hypothesis of whether species composition varied across the size classes using the *mvabund* package (Wang, Naumann, Wright, & Warton, 2012) in R considering sample sizes as offset (family: negative binomial) and significance was evaluated with an *anova* test (*manyglm*; resampling method “montecarlo,” number of bootstrap: 10,000) correcting *p*‐values for multiple comparisons (*p.adjust* method).

The food web representation was performed using Gephi software (https://gephi.org), which also contains routines for calculation of basic network indices such as degree (number of links per node) and other measures of centrality used to better represent the web of links (Cherven, 2013).

Indicator species analysis was performed to determine which OTU had significantly different frequency among *M. merluccius* size‐classes. The analysis was performed using the “*signassoc”* function in the “*indicspecies”* R package (Cáceres & Legendre, 2009) on both occurrence and relative abundance (ORA) data. We used mode = 1 (group‐based) and reported p‐values after Sidak's correction for multiple testing. Moreover, the function *multipatt* was used for determining lists of species that are associated to particular groups of sites (or combinations of those). Prey‐specific abundance (PSA), a function of the percentage of prey items in only those stomachs in which the prey occurs, was calculated according to the following formula

$$P_{i}=\sum S_{i}\cdot {\left(\sum S_{\mathrm{ti}}\right)}^{-1}\cdot 100$$

where *P*_*i*_ is the prey‐specific abundance of prey *i*,* S*_*i*_ the stomach content (number) comprising prey *i* and *S*_*ti*_ the total stomach content in only those predators with prey *i* in their stomach (Amundsen, Gabler, & Staldvik, 1996). The PSA index was computed on both ORA data and morphological data considering only prey detected at the species level in both the analyses (namely *Alpheus glaber*,* Engraulis encrasicolus* and *Solenocera membranacea*).

To evaluate if prey abundance in the diet of *M. merluccius* can be correlated to the abundance of the prey species in the North‐Central Adriatic sampling area, we plotted the number of hake stomachs containing *E. encrasicolus* vs the abundance of *E. encrasicolus* estimated during MEDITS 2014 survey for the same hauls.

Illumina DNA sequences obtained during the current study were deposited in the ENA's Sequence Read Archive (http://www.ebi.ac.uk/ena) under the accession number PRJEB22703.

*Merluccius merluccius* is a commercial species; therefore, neither special permits nor ethics approval were required for their collection, stomach dissection was performed post mortem.

## 3 RESULTS

The two primers selected for the metabarcoding analysis of stomach contents showed a high coverage of taxa: 80,000 species were amplified and all the potential prey families among taxa were represented with >3,000 species by the in silico ecoPCR assay (Ficetola et al., 2010). Moreover, the in vitro PCR assays performed on tissue DNA of the most common *M. merluccius* prey (see Section 2 and Supporting Information Table S2) allowed us to set up thermal conditions in order to obtain a good amplification efficiency of all the prey species targeted (Supporting Information Figure S1).

The results obtained from the identification of species in the positive control allowed us to identify all the species present in the mock sample with the exception of *Lophogaster typicus* which is absent in the GeneBank; moreover, for both *Alloteuthis* and *Sepietta* species, we could assign the sequences only at the genus level (Supporting Information Figure S2) because of a lack of differentiation among species within the DNA fragment used. Interestingly we were able to detect also single species with a very low amplification success (Supporting Information Figure S1). Moreover, the number of OTUs assigned to each individual ranged from 1 to 30 with most of the species ranging between 1 and 5 OTUs per individual.

The similarity search analysis of stomach content DNA sequences carried out against the GenBank nucleotide collection (nr/nt), detected 34 prey at the species level, in spite of the only eight species detected by the morphological identification and all the other items were classified at higher taxonomic rank (e.g., Teleostei, Table 1). Moreover, when considering a total of 200 stomachs analyzed using the morphological method, items classified at the species level raised to 11 and 5 were classified only at the genus level while all other items were classified at higher taxonomic rank.

**Table 1:** Species identified in *M. merluccius* stomach contents with both the metabarcoding and morphological approaches

Identified species	Number of stomachs	
	Molecular results	Morphological results
*Allotheutis* sp.	0	1
*Alpheus glaber*	14	7
*Anisakis pegreffii*	1	0
*Arnoglossus* sp.	1	0
*Chlorotocus crassicornis*	1	0
*Citharus linguatula*	1	0
Decapoda	0	14
*Eledone moschata*	1	0
*Engraulis encrasicolus*	44	8
*Gaidropsarus mediterraneus*	1	0
*Gobius niger*	0	3
*Holothuria forskali*	1	0
*Illex coindetii*	1	0
*Lesueurigobius friesii*	14	0
*Liocarcinus depurator*	1	0
*Melicertus kerathurus*	1	0
*Merlangius merlangus*	1	0
*Microchirus variegatus*	1	0
*Mullus barbatus*	4	0
*Mullus surmuletus*	2	0
*Pagellus acarne*	2	0
*Pagellus* sp.	0	1
*Philocheras bispinosus*	4	0
*Pleurobranchaea meckeli*	1	0
*Processa modica*	1	0
*Processa nouveli*	26	0
*Processa* sp.	0	5
*Raja miraletus*	2	0
*Rissoides desmaresti*	0	1
*Sardina pilchardus*	5	0
*Scomber colias*	1	0
*Scophthalmus maximus*	2	0
*Scorpaena notata*	1	0
*Sepia officinalis*	1	0
*Serranus hepatus*	1	0
*Solenocera membranacea*	24	2
*Spicara maena*	3	0
*Trachurus mediterraneus*	2	0
*Trachurus trachurus*	2	0
Teleostei	0	19
*Upogebia deltaura*	1	0

**Figure 3:**
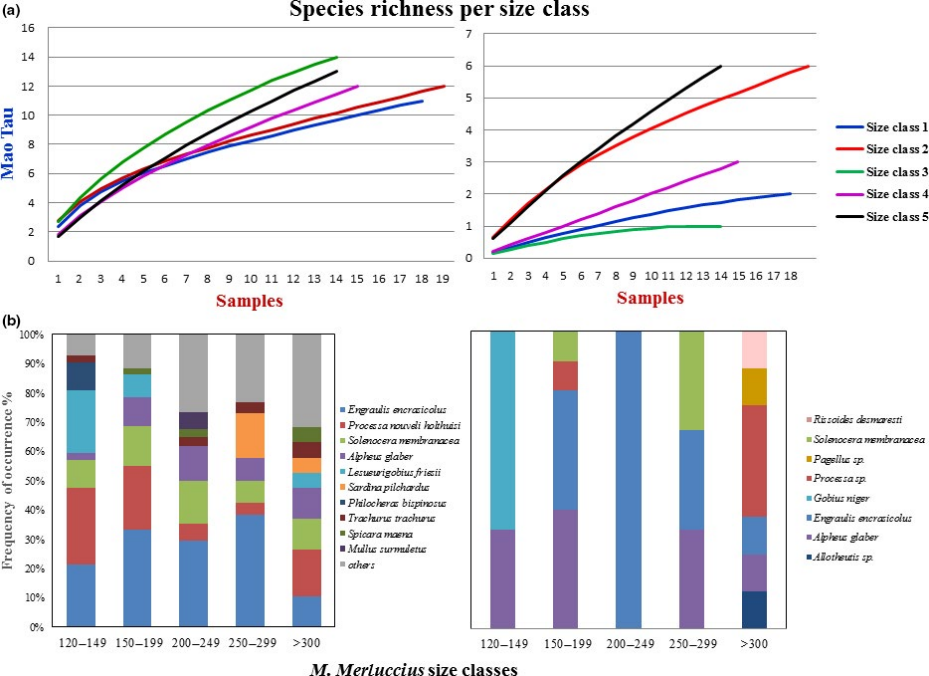
Comparison of dietary richness among *M. merluccius* size classes. (a) Sample‐based species richness curves for each size class for the metabarcoding data (left) and morphological data (right); (b) Main prey of *M. merluccius* by size classes as identified by metabarcoding approach (left) and morphological data (right). Frequency occurrence data of species are reported. The 10 most recurrent items across all classes are showed

The adequacy of stomach sample sizes (Figure 3a, left) was assessed by generating accumulation curves (with 1,000 random iterations) of species recorded per stomach sample. Because none of the accumulation curves reached a stable plateau, the nonparametric Incidence‐based Coverage Estimator (Foggo, Attrill, Frost, & Rowden, 2003) was used to estimate total dietary richness. The identified species accounted for approximately 53% of the theoretical plateau, that is, the richness estimated at the upper limit of sampling effort. The same analysis performed using the morphological identification results (Figure 3a, right) highlighted an even stronger underestimation of the species richness for all the size classes, showing values of richness ten times lower than the values obtained by using the metabarcoding approach. Only when using a higher number of stomachs for the morphological analysis (a total of 200, Supporting Information Figure S3a), the species richness increased up to 12 only for the larger size, but overall the values were still not comparable to metabarcoding accumulation curve. The ranking order by occurrence obtained with the metabarcoding data of the first 10 species (Figure 3b, left) highlighted that they constitute from 92% (smaller size class) to 70% (larger size class) of the total species identified in the *M. merluccius* size classes. In particular, one teleost (*E. encrasicolus*) and 3 decapods (*Processa nouveli holthuisi*,* S. membranacea,* and *A. glaber*) are recurrent in all size classes and constitute >50% of the species identified with the metabarcoding approach. Other species (e.g., *Lesueurigobius friesii* and *Philocheras bispinosus*) showed higher frequency or were restricted to only one class (Total Length, TL 120–149 mm). The frequency of other items (out of the 10 most recurrent) is increasing with size indicating an increase in the spectrum of prey. The morphological results (Figure 3b, right) allowed the identification of a very limited spectrum of prey as expected by the accumulation curves and showed an overall lower richness of prey species compared to the metabarcoding approach. The morphological analysis performed using 200 samples showed a higher number of prey detected in comparison with the same analysis performed on 95 stomachs; however, we could appreciate this improvement only when considering also prey identified at the genus level (Supporting Information Figure S3a, i.e., species identified as spp.). Notably, the morphological analyses highlighted a higher prey diversity for the larger size class as for the metabarcoding results.

**Figure 4:**
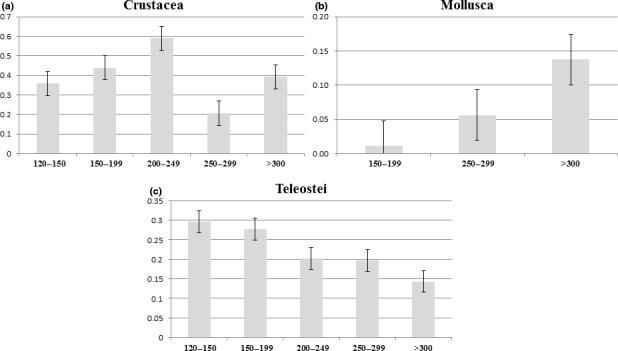
Comparison of mean ORA for each size class. Mean ORA (±*SD*) per sample for (a) Crustacea, (b) Mollusca and (c) Teleostei. No significant comparison was revealed after Kruskall‐Wallis test (Kruskal & Wallis, 1952) (*p*‐value = >0.05)

The OTUs Relative Abundance (ORA) of the three main dietary taxa (Crustacea, Teleostei, Mollusca) across *M. merluccius* size classes are compared in Figure 4. In general, this analysis highlighted a slight preference for crustaceans for the size classes 2 and 3 (TL 150–199 mm and 200–249, Figure 4a), confirmed the higher abundance of molluscs in the diet of the largest size class (Figure 4b), and a greater mean ORA of teleosts for the smaller size class (TL 120–149 mm, Figure 4c). The Kruskall–Wallis test (Kruskal & Wallis, 1952) did not find any significant comparisons among the ORA of the five size classes for teleosts and crustaceans (*p*‐value > 0.05), while the number of molluscs families was too low to consider the test reliable.

Bray‐Curtis dietary dissimilarities ranged between 0.351(size class TL 150–199 vs. TL 200–249) and 0.616 (size class TL 120–149 vs. TL 250–299) for relative occurrence data and 0.31 (size class TL 150–199 vs. TL 200–249) and 0.70 (size class TL 120–149 vs. TL ≥300) for ORA data. The Wilcoxon signed‐rank test (Wilcoxon, 1945) was not significant suggesting that both indices provided consistent measures with regard to dietary niche partitioning (Table 2). In general, the dissimilarities showed intermediate values suggesting a partial dietary overlap among the five size classes of *M. merluccius*.

**Table 2:** Inter‐size class variability measured as Bray‐Curtis dissimilarities calculated using occurrence‐ (below diagonal) and ORA (above diagonal)‐based data

*M. merluccius* classes	Class = 120–149	Class = 150–199	Class = 200–249	Class = 250–299	Class ≥ 300
Class = 120–149		0.359	0.411	0.49	0.702
Class = 150–199	0.404		0.312	0.38	0.624
Class = 200–249	0.586	0.351		0.438	0.623
Class = 250–299	0.616	0.456	0.392		0.642
Class ≥ 300	0.613	0.483	0.498	0.517	

**Figure 5:**
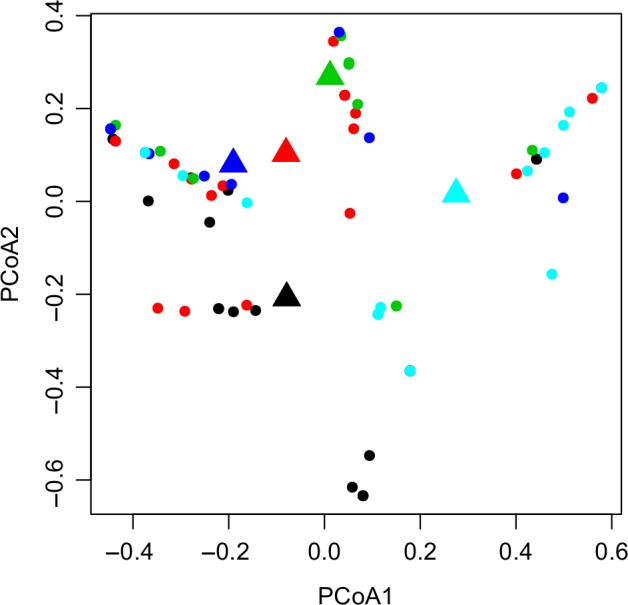
Principal Component Analysis. PCoA of relative occurrence‐based Bray‐Curtis dissimilarity of samples from all size classes (permutest *p*‐value = 0.017). Triangles depict the centroids of the distributions. Black color: size class 1, red: size class 2, green: size class 3, blue: size class 4, light blue: size class 5

The principal component analysis (PCoA) plot, based on the Bray‐Curtis distances computed on the relative occurrence data (Figure 5), showed a significant (permutest *p*‐value = 0.017) partial clustering among the size classes and a moderate differentiation of size classes 2 and 5 (*p*‐value = 0.001) and classes 2 and 4 (*p*‐value = 0.027). This result suggested a general homogeneity of variance within the size classes also supported by the *anosim* analysis (*R* ^2^ = 0.22, *p*‐value = 0.00099). The low although significant value of the determination coefficient suggested a lack of discrimination between groups. Similarly the permutational (per)MANOVA analysis (*R* ^2^ = 0.19, *p*‐value = 0.00099) showed that only 19% of variance was explained by the tested groupings.

The application of GLM to test the multivariate hypothesis of whether species composition varied across classes resulted strongly significant (Likelihood Ratio Test = 180.5 *p*‐value = <2^e−16^) highlighting the presence of an effect of groups on species composition.

**Figure 6:**
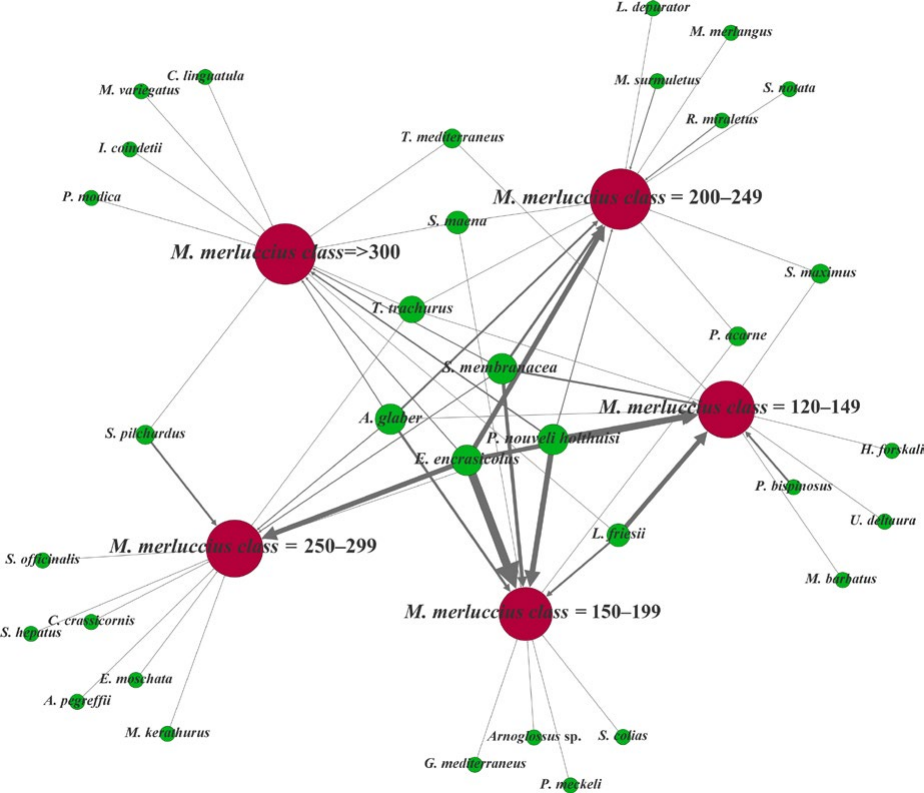
The food web related to the predator *M. merluccius*. Brown nodes indicate the predator divided into five size classes and green nodes represent the prey. The size of nodes is proportional to number of links connected (degree), and the size of links is proportional to the number of times the link prey–predator was found in the samples. Species are distributed according to their linkage with predator size classes: the prey species common to all size classes are in the middle

The food web network (Figure 6) allowed us to identify the number of prey shared by all the size classes and highlighted a high number of size‐specific prey. In spite of the similarities identified for the relative occurrences of the highest taxonomic class (Crustacea, Mollusca, Teleostei), the Bray‐Curtis dissimilarities showed differentiations among size classes and the GLM analysis clearly detected an effect of groups. In particular, the web of trophic interactions derived from metabarcoding approach showed that prey species shared by all size classes are *E. encrasicolus*,* P. nouveli holthuisi*,* S. membranacea*,* A. glaber* (Figure 6, but also Figure 3b). Other prey species, such as *Sardina pilchardus* and *L. friesii*, for example, tend to be preferred by large and small *M. merluccius* individuals, respectively. Moreover, the network highlighted the presence of clouds of size‐specific prey species which were detected only in one size class, displaying an extremely high complexity of the trophic interaction of European hake in the North‐Central Adriatic.

Both the *multipatt* and the *signassoc* functions performed on ORA data for identifying indicator species revealed a lower number of significant associations in comparison with sequence relative occurrence data (Supporting Information Tables S3 and S4) indicating that the relative abundance of OTUs provided the more conservative measure for our indicator‐species analysis. The *multipatt* function identified two species (*L. friesii* and *P. bispinosus*) significantly associated to the size class 1 (TL 120–149 mm) with high specificity and low sensitivity (Supporting Information Table S4) corroborating the food web network result (Figure 6). Moreover, *E. encrasicolus* showed a significant association with high specificity and sensitivity but this association concerned four hake size classes out of five suggesting that this prey is ubiquitous in the diet composition at least for the habitat and geographical area under study. The *signassoc* function confirmed this result also after correcting for multiple testing and highlighted the higher frequency of *L. friesii* and *P. bispinosus* in the size class 1 (Supporting Information Table S3).

**Figure 7:**
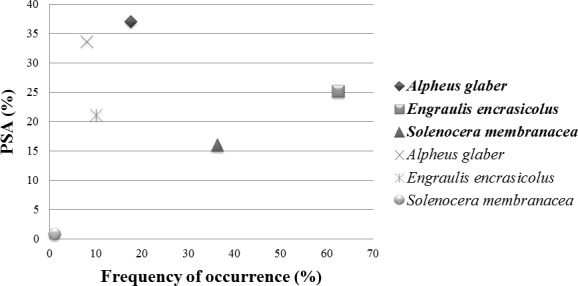
Feeding strategy diagram. Prey‐specific abundance (PSA) plotted against frequency of occurrence of prey. Only the species identified with both the metabarcoding and morphological analyses were considered. In bold the species found with the metabarcoding approach

The diet of *M. merluccius* in the North‐Central Adriatic did not show any evidence for specialization using prey‐specific abundance index (Amundsen et al., 1996; PSA < 0.5; Figure 7) computed both on metabarcoding and morphological data, and the highest value of PSA is obtained for *A. glaber* (0.37). This analysis showed a good agreement of PSA values obtained with the two methods of taxonomic identification and the relationship of PSA and frequency of occurrence suggested a broad niche width and low specialization for *M. merluccius*.

**Figure 8:**
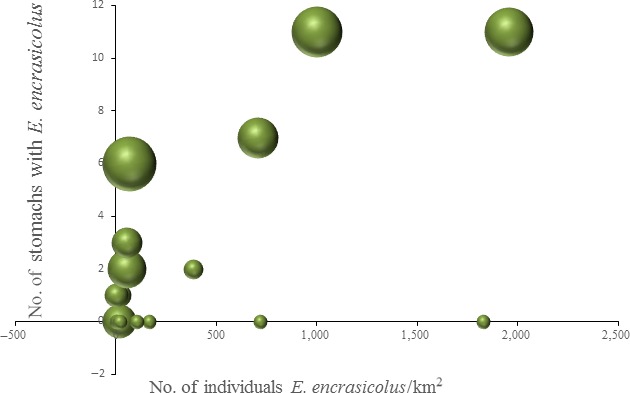
Prey–predator functional relationship. Number of *M. merluccius* stomachs containing *E. encrasicolus* in relation to the abundance of *E. encrasicolus* estimated for the same hauls (data from MEDITS 2014 survey). The bubbles size is proportional to the number of *M. merluccius* stomach data (specimen, e.g., number of individuals) available per haul

Finally we found a good correlation between the number of stomachs containing *E. encrasicolus* and the estimated abundance of this species in the same hauls obtained using MEDITS 2014 survey data (Figure 8), confirming the opportunistic feeding strategy of *M. merluccius*.

## 4 DISCUSSION

In this study, we developed a metabarcoding method based on COI PCR amplification to evaluate its efficiency for the analysis of European hake diet and to increase our knowledge about its feeding habits and trophic relationships. The comparison of the molecular and morphological results clearly showed that the metabarcoding approach consistently detected a wider spectrum of prey species than classical approach, providing a thorough description of *M. merluccius* diet and trophic links.

The wide range of habitat and the different biocenosis sampled allowed us to characterize *M. merluccius* overall feeding strategy in the North‐Central Adriatic, that revealed to be very diverse across sizes and sites. Unfortunately our sample did not include the Adriatic nursery area of Pomo/Jabuka pit, were the youngest individuals (TL < 120 mm, size class 0) usually live during the juvenile phase, preventing the detection of the characteristic ontogenetic shift in diet (Carpentieri et al., 2005). Juvenile hakes (TL < 120 mm) are almost restricted to this area as a consequence of their limited mobility and usually their diet is quite different from adults because of their smaller size and the different environment inhabited (between 100 and 200 m depth). Metabarcoding results in terms of richness, however, highlighted that the rarefaction curve did not reach the theoretical plateau, suggesting the need of a higher number of individuals to be sampled in future analyses. Nevertheless, these preliminary results provide a general description of the trophic preferences of this voracious predator within the North‐Central Adriatic basin and, particularly, highlighted the higher efficiency of the DNA‐based method in detecting prey compared to the classical morphological approach. For instance, metabarcoding outperformed the morphological method in identifying prey not only on the same sample size, but even when using a number of stomachs as high as 200. Furthermore, our metabarcoding results on 95 individuals allowed the identification of 34 species in the diet getting closer to a previous extensive study on European hake diet (Carpentieri et al., 2005) which allowed the identification of 46 prey species on the basis of the morphological analysis of a very high number of hake stomachs (2761). These considerations highlight the much higher efficiency of the metabarcoding approach, especially because we used a very conservative approach for the assignment of OTUs considering that only OTUs with a similarity ≥97% were assigned with confidence to a species and rejecting sequences assigned only at the genus level.

Unexpectedly our analysis highlighted significant statistical differences also among the larger size classes (>120 mm) suggesting a general opportunistic feeding behavior and the presence of some kind of differentiation when considering the single prey species as highlighted by the food network analysis. Overall our metabarcoding results describe a diet based mainly on crustaceans and teleosts with a slightly higher abundance of molluscs detected in the *M. merluccius* of the larger size. The low number of stomachs containing molluscs, notably cephalopods, can be attributed to the low depth of the sampling sites (<150 m), and the sub‐area of recruitment (North‐Central Adriatic) as these factors can affect the variation of the abundance of these species (Krstulovic Sifner et al., 2005). Despite an overall homogeneous composition of *M. merluccius* diet, there were some indicator species that were distinctive in the size class formed by the youngest individuals (TL 120–149 mm), namely *L. friesii* and *P. bispinosus*. Moreover, a clear expansion of the spectrum of prey was visible when the size of individuals increases, suggesting a reduced selectivity of the largest *M. merluccius*. On the other side, the largest size showed a clear decrease of teleosts in diet and a sensible increase in crustaceans and molluscs that is coherent with other results based on larger samples (Carpentieri et al., 2005) but is not clearly highlighted by our morphological data.

Interestingly, measures of dietary dissimilarities obtained with both relative occurrence and ORA data were comparable suggesting a good performance of relative abundances in describing sample diversity. Indeed our mock sample showed that each individual can be represented by a limited number of OTUs and, with the exception of few species showing more than ten OTUs per individuals (Supporting Information Figure S2), all the others were represented by a very low number of OTUs. The identification of a cloud of OTUs for each individual can be associated with both the presence of pseudogenes/intra‐individual polymorphisms that are intrinsic features of the biological complexity of genomes, and sequencing artifacts that are a well‐known limit of high‐throughput sequencing and that can be overcome using rigorous methods of analysis. The PSA analysis showed a good concordance of values obtained with both the metabarcoding and morphological methods, suggesting that, despite the semi‐quantitative nature of metabarcoding analysis of stomach content, this technique can indeed describe faithfully the diet of European hake.

In addition, the high frequency of occurrence detected for anchovy can be related to a high number of anchovies present in the area during the campaign or a high species abundance in the sampled area.

Remarkably the plot of the number of stomachs containing *E. encrasicolus* and the abundance obtained from MEDITS 2014 survey corroborated this evidence, showing a good concordance between the presence in the diet and the abundance of this species in the sampling area. The relationship found resemble the typical prey–predator functional response (Holling, 1959) that is largely applied in trophic ecology suggesting also the potential semi‐quantitative use of the metabarcoding results in dynamic trophic models (see, e.g., Angelini et al., 2016). Our analyses were able to describe in detail the diet of European hake, and the comparison with the classical method showed that the diet detail gained with metabarcoding approach was impossible to reproduce with the morphological data obtained from the same samples. The metabarcoding approach presented here is thus very promising for a faithful description of the food network, which is a crucial task in the context of fisheries management. There is evidence, in fact, that the increase/decrease in key predators that are often targets for exploitation can have strong effects on prey and on the whole ecosystem (Baum & Worm, 2009; Heithaus, Frid, Wirsing, & Worm, 2008; Worm & Myers, 2003). The predatory effects can propagate down to the primary producers of the food web in the so called “trophic cascade,” with possible impact on species that has fundamental role in maintaining the ecosystem functionality (e.g., Myers, Baum, Shepherd, Powers, & Peterson, 2007). Several works (Mackinson et al., 2009; Stäbler et al., 2016; Walters et al., 2005) highlighted that effects of fisheries management can propagate through the food web with possible important unexpected feedbacks and thus optimal management requires a better disentanglement of trophic interactions, especially in the case of mixed fisheries. Furthermore, the removal of keystone predators causes a loss of species diversity at trophic levels lower in the food web (Paine, 1966); therefore, the knowledge of the food chain of predators of commercial interest is decisive for a sustainable management of fisheries.

Although DNA molecular data are unable, so far, to provide information about volumes or weights of ingested prey, here we showed that the metabarcoding approach can provide a new complementary basis to morphological and stable isotope approaches for further improvement of actual knowledge on feeding preferences.

In conclusion, although still preliminary our study highlights the exceptional potential of metabarcoding as an approach to provide unprecedented taxonomic resolution in the diet of *M. merluccius*. These data represent an important basis to reconstruct marine food webs and provide crucial insights for a sustainable management of this precious fishery resource.

## CONFLICT OF INTERESTS

The authors declare no competing financial interests.

## AUTHOR CONTRIBUTIONS

G.R., S.L., C.P. conceived the project and designed research; G.R. and M.S. performed research; G.R. and S.L. analyzed data; G.R. and S.L. wrote the paper; C.P. provided laboratory space, instrumentation and funding; S.L provided funding. All authors reviewed and approved the final manuscript.

## DATA ACCESSIBILITY

Illumina DNA sequences obtained during the current study were deposited in the ENA's Sequence Read Archive (http://www.ebi.ac.uk/ena) under the accession number PRJEB22703.
