# Supplementary material for: A metabarcoding approach for the feeding habits of European hake in the Adriatic Sea
Source: Ecol Evol. 2018 Oct 6;8(21):10435–47. doi: 10.1002/ece3.4500 (PMC6238138; doi:10.1002/ece3.4500)
Supplement: Supplementary file 1 [file ECE3-8-10435-s001.doc]

**Supporting information for:**

**A metabarcoding approach for the feeding habits of European hake in the Adriatic Sea**

Giulia Riccioni^1^, Marco Stagioni^1^, Corrado Piccinetti^1^, Simone Libralato^2^

^1^Department of Biological, Geological and Environmental Sciences, University of Bologna, Italy.

^2^National Institute of Oceanography and Experimental Geophysics, Borgo Grotta Gigante 42/c, Sgonico, Italy.

Corresponding author:

Giulia Riccioni

Email: giulia.riccioni@unibo.it

**This file contains four Supplementary Tables and three Supplementary Figures.**

**Table S1.** Summary of sampling information: size class, geographical coordinates, haul number and depth of sampled specimens used for molecular analysis.

| **ID** | **Size class** | **Latitude (N)** | **Longitude (E)** | **Haul number** | **Depth** |
| --- | --- | --- | --- | --- | --- |
| sample_37 | 120-149 | 43°15.94’ | 14°06.15’ | 16 | 60 |
| sample_38 | 120-149 | 43°15.94’ | 14°06.15’ | 16 | 60 |
| sample_39 | 120-149 | 43°15.94’ | 14°06.15’ | 16 | 60 |
| sample_40 | 120-149 | 43°15.94’ | 14°06.15’ | 16 | 60 |
| sample_41 | 120-149 | 43°15.94’ | 14°06.15’ | 16 | 60 |
| sample_42 | 120-149 | 43°15.94’ | 14°06.15’ | 16 | 60 |
| sample_43 | 120-149 | 43°15.94’ | 14°06.15’ | 16 | 60 |
| sample_33 | 150-199 | 43°15.94’ | 14°06.15’ | 16 | 60 |
| sample_35 | 150-199 | 43°15.94’ | 14°06.15’ | 16 | 60 |
| sample_36 | 150-199 | 43°15.94’ | 14°06.15’ | 16 | 60 |
| sample_31 | 200-249 | 43°15.94’ | 14°06.15’ | 16 | 60 |
| sample_32 | 200-249 | 43°15.94’ | 14°06.15’ | 16 | 60 |
| sample_34 | 200-249 | 43°15.94’ | 14°06.15’ | 16 | 60 |
| sample_30 | 250-299 | 43°15.94’ | 14°06.15’ | 16 | 60 |
| sample_155 | 250-299 | 43°16.25’ | 14°25.74’ | 18 | 86 |
| sample_4 | 120-149 | 43°40.44’ | 13°45.65’ | 20 | 52 |
| sample_6 | 120-149 | 43°40.44’ | 13°45.65’ | 20 | 52 |
| sample_10 | 120-149 | 43°40.44’ | 13°45.65’ | 20 | 52 |
| sample_11 | 120-149 | 43°40.44’ | 13°45.65’ | 20 | 52 |
| sample_12 | 120-149 | 43°40.44’ | 13°45.65’ | 20 | 52 |
| sample_13 | 120-149 | 43°40.44’ | 13°45.65’ | 20 | 52 |
| sample_2 | 150-199 | 43°40.44’ | 13°45.65’ | 20 | 52 |
| sample_3 | 150-199 | 43°40.44’ | 13°45.65’ | 20 | 52 |
| sample_5 | 150-199 | 43°40.44’ | 13°45.65’ | 20 | 52 |
| sample_7 | 150-199 | 43°40.44’ | 13°45.65’ | 20 | 52 |
| sample_8 | 150-199 | 43°40.44’ | 13°45.65’ | 20 | 52 |
| sample_9 | 150-199 | 43°40.44’ | 13°45.65’ | 20 | 52 |
| sample_1 | 250-299 | 43°40.44’ | 13°45.65’ | 20 | 52 |
| sample_26 | 120-149 | 43°46.79’ | 14°33.93’ | 22 | 83 |
| sample_27 | 120-149 | 43°46.79’ | 14°33.93’ | 22 | 83 |
| sample_28 | 120-149 | 43°46.79’ | 14°33.93’ | 22 | 83 |
| sample_29 | 120-149 | 43°46.79’ | 14°33.93’ | 22 | 83 |
| sample_23 | 150-199 | 43°46.79’ | 14°33.93’ | 22 | 83 |
| sample_24 | 150-199 | 43°46.79’ | 14°33.93’ | 22 | 83 |
| sample_25 | 150-199 | 43°46.79’ | 14°33.93’ | 22 | 83 |
| sample_17 | 200-249 | 43°46.79’ | 14°33.93’ | 22 | 83 |
| sample_21 | 200-249 | 43°46.79’ | 14°33.93’ | 22 | 83 |
| sample_16 | 250-299 | 43°46.79’ | 14°33.93’ | 22 | 83 |
| sample_14 | ≥ 300 | 43°46.79’ | 14°33.93’ | 22 | 83 |
| sample_15 | ≥ 300 | 43°46.79’ | 14°33.93’ | 22 | 83 |
| sample_62 | 250-299 | 43°23.77’ | 15°04.02’ | 27 | 115 |
| sample_64 | ≥ 300 | 43°23.77’ | 15°04.02’ | 27 | 115 |
| sample_150 | 250-299 | 43°13.21’ | 15°01.97’ | 29 | 135 |
| sample_151 | 250-299 | 43°13.21’ | 15°01.97’ | 29 | 135 |
| sample_148 | ≥ 300 | 43°13.21’ | 15°01.97’ | 29 | 135 |
| sample_149 | ≥ 300 | 43°13.21’ | 15°01.97’ | 29 | 135 |
| sample_133 | 250-299 | 43°10.12’ | 14°58.98’ | 30 | 143 |
| sample_159 | ≥ 300 | 42°53.25’ | 14°07.66’ | 39 | 53 |
| sample_157 | ≥ 300 | 43°32.45’ | 14°13.24’ | 48 | 82 |
| sample_74 | ≥ 300 | 44°01.08’ | 13°08.15’ | 49 | 40 |
| sample_47 | 150-199 | 44°04.37’ | 13°25.78’ | 50 | 63 |
| sample_48 | 150-199 | 44°04.37’ | 13°25.78’ | 50 | 63 |
| sample_49 | 150-199 | 44°04.37’ | 13°25.78’ | 50 | 63 |
| sample_52 | 150-199 | 44°04.37’ | 13°25.78’ | 50 | 63 |
| sample_53 | 150-199 | 44°04.37’ | 13°25.78’ | 50 | 63 |
| sample_44 | 200-249 | 44°04.37’ | 13°25.78’ | 50 | 63 |
| sample_45 | 200-249 | 44°04.37’ | 13°25.78’ | 50 | 63 |
| sample_46 | 200-249 | 44°04.37’ | 13°25.78’ | 50 | 63 |
| sample_50 | 200-249 | 44°04.37’ | 13°25.78’ | 50 | 63 |
| sample_59 | 150-199 | 44°07.08’ | 13°29.23’ | 51 | 63 |
| sample_60 | 150-199 | 44°07.08’ | 13°29.23’ | 51 | 63 |
| sample_54 | 200-249 | 44°07.08’ | 13°29.23’ | 51 | 63 |
| sample_56 | 200-249 | 44°07.08’ | 13°29.23’ | 51 | 63 |
| sample_58 | 200-249 | 44°07.08’ | 13°29.23’ | 51 | 63 |
| sample_61 | ≥ 300 | 44°07.08’ | 13°29.23’ | 51 | 63 |
| sample_67 | 120-149 | 44°12.12’ | 13°24.92’ | 52 | 61 |
| sample_66 | 200-249 | 44°12.12’ | 13°24.92’ | 52 | 61 |
| sample_73 | 250-299 | 44°12.12’ | 13°24.92’ | 52 | 61 |
| sample_156 | ≥ 300 | 44°43.72’ | 13°22.65’ | 71 | 42 |
| sample_130 | 250-299 | 44°57.88’ | 13°01.00’ | 89 | 32 |
| sample_153 | 250-299 | 44°15.67’ | 13°57.35’ | 101 | 62 |
| sample_154 | 250-299 | 44°15.67’ | 13°57.35’ | 101 | 62 |
| sample_136 | 250-299 | 44°17.26’ | 14°11.61’ | 102 | 60 |
| sample_137 | ≥ 300 | 44°17.26’ | 14°11.61’ | 102 | 60 |
| sample_138 | ≥ 300 | 44°17.26’ | 14°11.61’ | 102 | 60 |
| sample_96 | 250-299 | 44°11.77’ | 14°05.94’ | 103 | 68 |
| sample_93 | ≥ 300 | 44°11.77’ | 14°05.94’ | 103 | 68 |
| sample_94 | ≥ 300 | 44°11.77’ | 14°05.94’ | 103 | 68 |
| sample_95 | ≥ 300 | 44°11.77’ | 14°05.94’ | 103 | 68 |
| sample_140 | 250-299 | 44°09.46’ | 13°53.28’ | 104 | 65 |

**Table S2.** Common European hake preys used for PCR thermal condition set up and positive control sample. All DNAs were diluted 1:100 and pooled to create the DNA control sample that was amplified by PCR by using the same molecular protocols used for hake stomach contents.

|  | **Family** | **Prey** | **DNA quantification** |
| --- | --- | --- | --- |
|  | Solenoceridae | *Solenocera membranacea* | 0.96 ng/μl |
|  | Lophogastridae | *Lophogaster typicus* | 0.96 ng/μl |
| Crustaceans | Pandalidae | *Chlorotocus crassicornis* | 1.76 ng/μl |
|  | Processidae | *Processa modica* | 1.28 ng/μl |
|  | Polybiidae | *Liocarcinus depurator* | 10.76 ng/μl |
|  | Engraulidae | *Engraulis encrasicolus* | 2 ng/μl |
|  | Cepolidae | *Cepola macrophthalma* | 0.96 ng/μl |
|  | Lotidae | *Gaidropsarus biscayensis* | 0.97 ng/μl |
|  | Gobiidae | *Gobius niger* | 0.6 ng/μl |
|  | Gadidae | *Trisopterus capelanus* | 1.56 ng/μl |
|  | Gadidae | *Merlangius merlangus* | 1.12 ng/μl |
|  | Gadidae | *Gadiculus argenteus* | 0.6 ng/μl |
| Actinopterygii | Merlucciidae | *Merluccius merluccius* | 8 ng/μl |
|  | Gobiidae | *Lesueurigobius friesii* | 1.6 ng/μl |
|  | Gadidae | *Micromesistius poutassou* | 1.6 ng/μl |
|  | Callionymidae | *Synchiropus phaeton* | 0.8 ng/μl |
|  | Callionymidae | *Callionymus maculatus* | 3.44 ng/μl |
|  | Sternoptychidae | *Maurolicus muelleri* | 0.97 ng/μl |
|  | Clupeidae | *Sardina pilchardus* | 3.88 ng/μl |
|  | Ommastrephidae | *Illex coindetii* | 5.84 ng/μl |
|  | Loliginidae | *Alloteuthis subulata* | 1.6 ng/μl |
|  | Loliginidae | *Alloteuthis media* | 2.04 ng/μl |
|  | Loliginidae | *Loligo vulgaris* | 13.16 ng/μl |
|  | Sepiolidae | *Sepiola robusta* | 0.96 ng/μl |
|  | Sepiolidae | *Sepietta oweniana* | 1.8 ng/μl |
| Molluscs | Sepiolidae | *Sepietta neglecta* | 7.12 ng/μl |
|  | Sepiolidae | *Sepietta obscura* | 1.7 ng/μl |
|  | Sepiolidae | *Rossia macrosoma* | 8.4 ng/μl |
|  | Sepiidae | *Sepia elegans* | 13.76 ng/μl |

**Table S3.** *signassoc* analysis results for sequence occurrence data and ORA data (psidak = p-values after Sidak’s correction for multiple testing).

| **Sequence occurrence data** | **1** | **2** | **3** | **4** | **5** | best | psidak | **ORA data** | **1** | **2** | **3** | **4** | **5** | best | psidak |
| --- | --- | --- | --- | --- | --- | --- | --- | --- | --- | --- | --- | --- | --- | --- | --- |
| *Alpheus glaber* | 0.92 | 0.35 | 0.08 | 0.7 | 0.73 | 3 | 0.33 | *Alpheus glaber* | 0.94 | 0.49 | 0.02 | 0.54 | 0.75 | 3 | 0.07 |
| *Anisakis pegreffii* | 1 | 1 | 1 | 0.18 | 1 | 4 | 0.62 | *Anisakis pegreffii* | 1 | 1 | 1 | 0.17 | 1 | 4 | 0.61 |
| *Arnoglossus* | 1 | 0.23 | 1 | 1 | 1 | 2 | 0.74 | *Arnoglossus* | 1 | 0.23 | 1 | 1 | 1 | 2 | 0.73 |
| *Chlorotocus crassicornis* | 1 | 1 | 1 | 0.18 | 1 | 4 | 0.63 | *Chlorotocus crassicornis* | 1 | 1 | 1 | 0.18 | 1 | 4 | 0.64 |
| *Citharus linguatula* | 1 | 1 | 1 | 1 | 0.18 | 5 | 0.64 | *Citharus linguatula* | 1 | 1 | 1 | 1 | 0.2 | 5 | 0.67 |
| *Eledone moschata* | 1 | 1 | 1 | 0.21 | 1 | 4 | 0.70 | *Eledone moschata* | 1 | 1 | 1 | 0.19 | 1 | 4 | 0.64 |
| *Engraulis encrasicolus* | 0.77 | 0 | 0.04 | 0.22 | 1 | 2 | 0.01 | *Engraulis encrasicolus* | 0.57 | 0.09 | 0.23 | 0.17 | 1 | 2 | 0.37 |
| *Gaidropsarus mediterraneus* | 1 | 0.24 | 1 | 1 | 1 | 2 | 0.74 | *Gaidropsarus mediterraneus* | 1 | 0.23 | 1 | 1 | 1 | 2 | 0.73 |
| *Holothuria forskali* | 0.22 | 1 | 1 | 1 | 1 | 1 | 0.71 | *Holothuria forskali* | 0.24 | 1 | 1 | 1 | 1 | 1 | 0.74 |
| *Illex coindetii* | 1 | 1 | 1 | 1 | 0.2 | 5 | 0.67 | *Illex coindetii* | 1 | 1 | 1 | 1 | 0.17 | 5 | 0.61 |
| *Lesueurigobius friesii* | 0 | 0.2 | 1 | 1 | 0.8 | 1 | 0.00 | *Lesueurigobius friesii* | 0 | 0.56 | 1 | 1 | 0.93 | 1 | 0.00 |
| *Liocarcinus depurator* | 1 | 1 | 0.14 | 1 | 1 | 3 | 0.52 | *Liocarcinus depurator* | 1 | 1 | 0.17 | 1 | 1 | 3 | 0.60 |
| *Melicertus kerathurus* | 1 | 1 | 1 | 0.18 | 1 | 4 | 0.63 | *Melicertus kerathurus* | 1 | 1 | 1 | 0.18 | 1 | 4 | 0.64 |
| *Merlangius merlangus* | 1 | 1 | 0.16 | 1 | 1 | 3 | 0.58 | *Merlangius merlangus* | 1 | 1 | 0.16 | 1 | 1 | 3 | 0.58 |
| *Microchirus variegatus* | 1 | 1 | 1 | 1 | 0.18 | 5 | 0.63 | *Microchirus variegatus* | 1 | 1 | 1 | 1 | 0.2 | 5 | 0.66 |
| *Mullus barbatus* | 1 | 0.69 | 0.09 | 1 | 0.52 | 3 | 0.36 | *Mullus barbatus* | 1 | 0.67 | 0.17 | 1 | 0.19 | 3 | 0.61 |
| *Mullus surmuletus* | 1 | 1 | 0.02 | 1 | 1 | 3 | 0.11 | *Mullus surmuletus* | 1 | 1 | 0.02 | 1 | 1 | 3 | 0.10 |
| *Pagellus acarne* | 1 | 0.42 | 0.1 | 1 | 1 | 3 | 0.42 | *Pagellus acarne* | 1 | 0.42 | 0.06 | 1 | 1 | 3 | 0.26 |
| *Philocheras bispinosus* | 0 | 1 | 1 | 1 | 1 | 1 | 0.01 | *Philocheras bispinosus* | 0 | 1 | 1 | 1 | 1 | 1 | 0.00 |
| *Pleurobranchaea meckeli* | 1 | 0.24 | 1 | 1 | 1 | 2 | 0.75 | *Pleurobranchaea meckeli* | 1 | 0.25 | 1 | 1 | 1 | 2 | 0.76 |
| *Processa modica* | 1 | 1 | 1 | 1 | 0.19 | 5 | 0.65 | *Processa modica* | 1 | 1 | 1 | 1 | 0.19 | 5 | 0.66 |
| *Processa nouveli holthuisi* | 0 | 0.01 | 0.92 | 0.99 | 0.86 | 1 | 0.01 | *Processa nouveli holthuisi* | 0.25 | 0.31 | 0.14 | 1 | 0.7 | 3 | 0.54 |
| *Raja miraletus* | 1 | 1 | 0.03 | 1 | 1 | 3 | 0.13 | *Raja miraletus* | 1 | 1 | 0.03 | 1 | 1 | 3 | 0.13 |
| *Sardina pilchardus* | 1 | 1 | 1 | 0 | 0.64 | 4 | 0.01 | *Sardina pilchardus* | 1 | 1 | 1 | 0.04 | 0.22 | 4 | 0.19 |
| *Scomber colias* | 1 | 0.24 | 1 | 1 | 1 | 2 | 0.75 | *Scomber colias* | 1 | 0.25 | 1 | 1 | 1 | 2 | 0.76 |
| *Scophthalmus maximus* | 0.44 | 1 | 0.17 | 1 | 1 | 3 | 0.60 | *Scophthalmus maximus* | 0.4 | 1 | 0.09 | 1 | 1 | 3 | 0.38 |
| *Scorpaena notata* | 1 | 1 | 0.18 | 1 | 1 | 3 | 0.62 | *Scorpaena notata* | 1 | 1 | 0.15 | 1 | 1 | 3 | 0.57 |
| *Sepia officinalis* | 1 | 1 | 1 | 0.19 | 1 | 4 | 0.66 | *Sepia officinalis* | 1 | 1 | 1 | 0.19 | 1 | 4 | 0.64 |
| *Serranus hepatus* | 1 | 1 | 1 | 0.18 | 1 | 4 | 0.64 | *Serranus hepatus* | 1 | 1 | 1 | 0.2 | 1 | 4 | 0.67 |
| *Solenocera membranacea* | 0.99 | 0.24 | 0.03 | 0.98 | 0.25 | 3 | 0.16 | *Solenocera membranacea* | 0.84 | 0.64 | 0.38 | 0.72 | 0.06 | 5 | 0.26 |
| *Spicara maena* | 1 | 0.56 | 0.27 | 1 | 0.36 | 3 | 0.79 | *Spicara maena* | 1 | 0.57 | 0.31 | 1 | 0.15 | 5 | 0.56 |
| *Trachurus mediterraneus* | 0.32 | 1 | 1 | 1 | 0.21 | 5 | 0.69 | *Trachurus mediterraneus* | 0.18 | 1 | 1 | 1 | 0.24 | 1 | 0.62 |
| *Trachurus trachurus* | 0.63 | 1 | 0.5 | 0.51 | 0.51 | 3 | 0.97 | *Trachurus trachurus* | 0.46 | 1 | 0.4 | 0.56 | 0.17 | 5 | 0.61 |
| *Upogebia deltaura* | 0.23 | 1 | 1 | 1 | 1 | 1 | 0.73 | *Upogebia deltaura* | 0.25 | 1 | 1 | 1 | 1 | 1 | 0.76 |

**Table S4.** *multipatt* analysis results for ORA data for the five size classes.

| **Multipatt analysis, association species/sites with ORA data** |  |  |  |  |  |  |  |  |
| --- | --- | --- | --- | --- | --- | --- | --- | --- |
|  | **s.1** | **s.2** | **s.3** | **s.4** | **s.5** | **index** | **stat** | **p-value** |
| *Alpheus glaber* | 0 | 1 | 1 | 1 | 1 | 30 | 0.4333648 | 0.435 |
| *Anisakis pegreffii* | 0 | 0 | 0 | 1 | 0 | 4 | 0.2581989 | 0.537 |
| *Arnoglossus* | 0 | 1 | 0 | 0 | 0 | 2 | 0.2294157 | 1.000 |
| *Chlorotocus crassicornis* | 0 | 0 | 0 | 1 | 0 | 4 | 0.2581989 | 0.552 |
| *Citharus linguatula* | 0 | 0 | 0 | 0 | 1 | 5 | 0.2581989 | 0.536 |
| *Eledone moschata* | 0 | 0 | 0 | 1 | 0 | 4 | 0.2581989 | 0.520 |
| *Engraulis encrasicolus* | 1 | 1 | 1 | 1 | 0 | 26 | 0.8263713 | 0.001 |
| *Gaidropsarus mediterraneus* | 0 | 1 | 0 | 0 | 0 | 2 | 0.2294157 | 1.000 |
| *Holothuria forskali* | 1 | 0 | 0 | 0 | 0 | 1 | 0.2357023 | 0.760 |
| *Illex coindetii* | 0 | 0 | 0 | 0 | 1 | 5 | 0.2581989 | 0.534 |
| *Lesueurigobius friesii* | 1 | 0 | 0 | 0 | 0 | 1 | 0.6367716 | 0.001 |
| *Liocarcinus depurator* | 0 | 0 | 1 | 0 | 0 | 3 | 0.2773501 | 0.164 |
| *Melicertus kerathurus* | 0 | 0 | 0 | 1 | 0 | 4 | 0.2581989 | 0.552 |
| *Merlangius merlangus* | 0 | 0 | 1 | 0 | 0 | 3 | 0.2773501 | 0.170 |
| *Microchirus variegatus* | 0 | 0 | 0 | 0 | 1 | 5 | 0.2581989 | 0.551 |
| *Mullus barbatus* | 0 | 0 | 1 | 0 | 1 | 14 | 0.3201856 | 0.346 |
| *Mullus surmuletus* | 0 | 0 | 1 | 0 | 0 | 3 | 0.3922323 | 0.026 |
| *Pagellus acarne* | 0 | 0 | 1 | 0 | 0 | 3 | 0.2597676 | 0.360 |
| *Philocheras bispinosus* | 1 | 0 | 0 | 0 | 0 | 1 | 0.4714045 | 0.004 |
| *Pleurobranchaea meckeli* | 0 | 1 | 0 | 0 | 0 | 2 | 0.2294157 | 1.000 |
| *Processa modica* | 0 | 0 | 0 | 0 | 1 | 5 | 0.2581989 | 0.532 |
| *Processa nouveli holthuisi* | 1 | 1 | 1 | 0 | 0 | 16 | 0.6498356 | 0.033 |
| *Raja miraletus* | 0 | 0 | 1 | 0 | 0 | 3 | 0.3922323 | 0.021 |
| *Sardina pilchardus* | 0 | 0 | 0 | 1 | 1 | 15 | 0.4082483 | 0.049 |
| *Scomber colias* | 0 | 1 | 0 | 0 | 0 | 2 | 0.2294157 | 1.000 |
| *Scophthalmus maximus* | 1 | 0 | 1 | 0 | 0 | 7 | 0.2540003 | 0.466 |
| *Scorpaena notata* | 0 | 0 | 1 | 0 | 0 | 3 | 0.2773501 | 0.153 |
| *Sepia officinalis* | 0 | 0 | 0 | 1 | 0 | 4 | 0.2581989 | 0.542 |
| *Serranus hepatus* | 0 | 0 | 0 | 1 | 0 | 4 | 0.2581989 | 0.547 |
| *Solenocera membranacea* | 0 | 1 | 1 | 1 | 1 | 30 | 0.6192578 | 0.245 |
| *Spicara maena* | 0 | 0 | 1 | 0 | 1 | 14 | 0.2585022 | 0.622 |
| *Trachurus mediterraneus* | 1 | 0 | 0 | 0 | 1 | 9 | 0.2461830 | 0.710 |
| *Trachurus trachurus* | 1 | 0 | 1 | 1 | 1 | 29 | 0.2560738 | 0.844 |
| *Upogebia deltaura* | 1 | 0 | 0 | 0 | 0 | 1 | 0.2357023 | 0.770 |

**Fig. S1.** Agarose gel image showing the amplification success of a 313bp COI fragment across 31 European hake preys. The forward primer mlCOIintF and reverse primer jgHCO2198 were used.

**
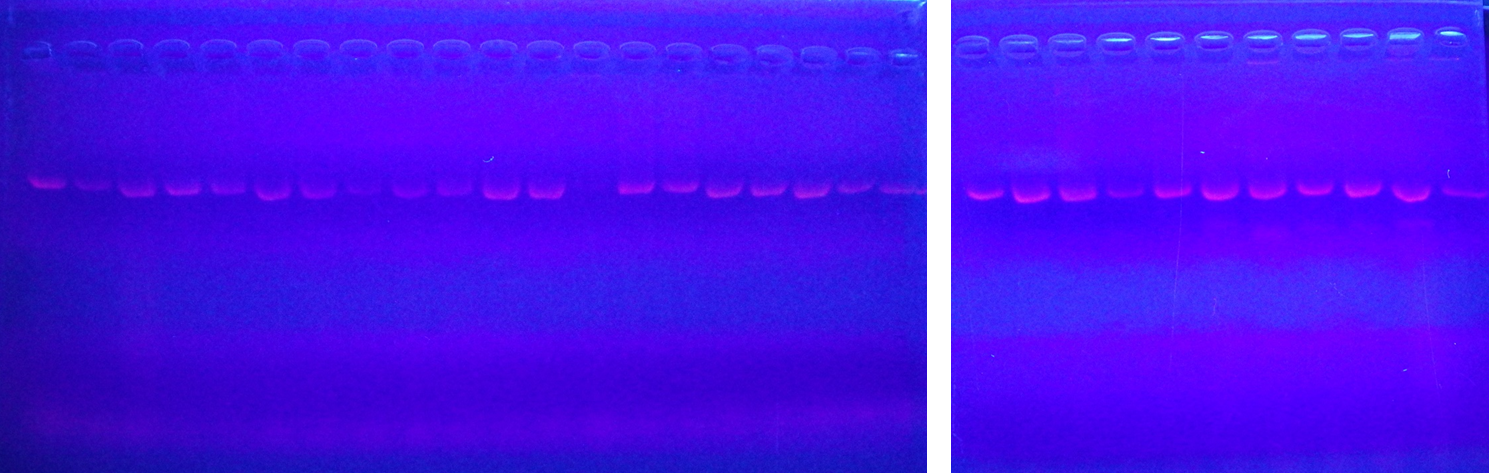
**

**Fig. S2.** Positive control sample result. The distribution of the number of OTUs for each species is shown.

**
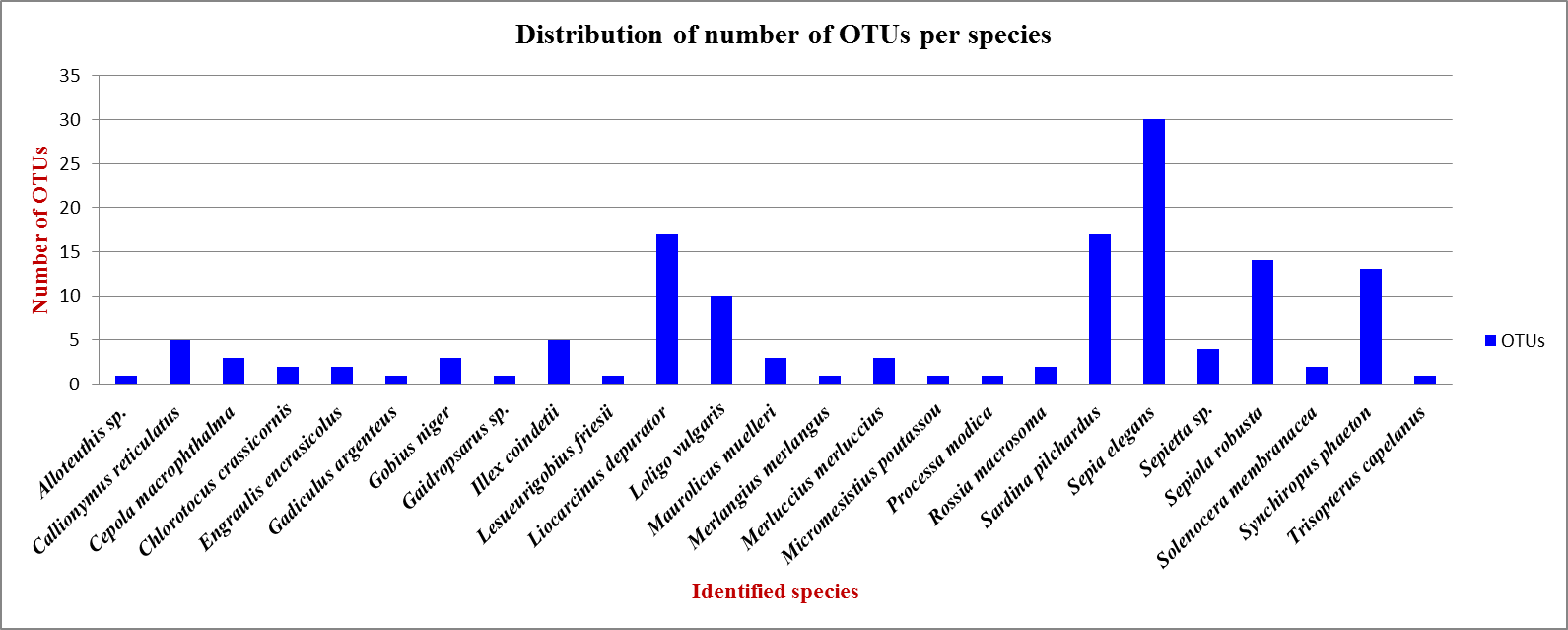
**

**Fig. S3.** Dietary richness among *M. merluccius* size classes for the 200 stomachs sample. a) Sample-based species richness curve for each size class for morphological data obtained by 200 stomachs; b) Main preys of *M. merluccius* by size classes as identified by the morphological data. Frequency occurrence data of species are reported. The ten most recurrent items across all classes are showed.

**
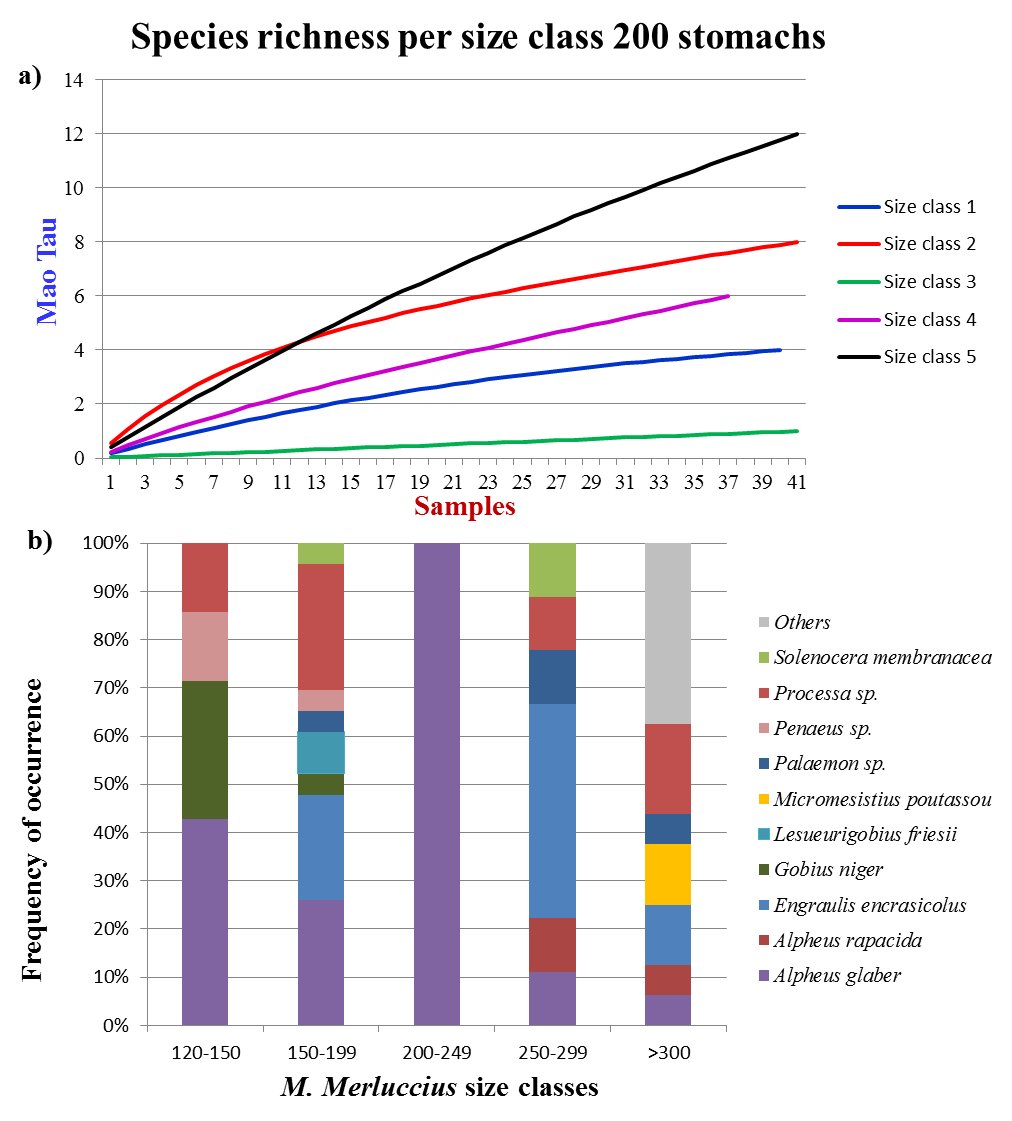
**
